# Experimental Ultrasound Transmission through Fluid-Solid and Air-Solid Phononic Plates

**DOI:** 10.3390/ma9060453

**Published:** 2016-06-07

**Authors:** Vicente Gómez-Lozano, Constanza Rubio, Pilar Candelas, Antonio Uris, Francisco Belmar

**Affiliations:** Centro de Tecnologías Físicas, Universitat Politécnica de Valencia, Avd. Los Naranjos, s/n., Valencia 46022, Spain; vgomez@fis.upv.es (V.G.-L.); pcandelas@fis.upv.es (P.C.); auris@fis.upv.es (A.U.); fbelmar@fis.upv.es (F.B.)

**Keywords:** subwalength hole array, perforated plate, phononic plate, Lamb waves, 43.20.+g, 43.35.+d, 42.79.Dj

## Abstract

Underwater ultrasonic transmissions for fluid-solid and air-solid phononic brass plates are reported in this work. Although the structure is roughly the same, experimental results show very different behaviour between fluid-solid and air-solid phononic plates, due to most of the properties of the fluid-solid perforated plates rely on Fabry-Perot resonances, Wood anomalies and Lamb modes. In air-solid phononic plates Fabry-Perot resonance is highly attenuated due to impedances difference between air and water, and therefore some transmission modes are now distinguishable due to surface modes coupling.

## 1. Introduction

At the end of the 19th century Lord Rayleigh [1] studied the reflection coefficient of a one-dimensional grating. This was the first study of sound interaction with periodic structures. Since then there have been major studies of sound wave propagation through periodic structures. During the last decades, much attention has been paid to phononic crystal. The beginning of the research in the field of phononic crystals started nearly two decades ago considering mainly bulk wave propagation [2,3]. Concepts such as band-gaps, waveguiding, and negative refraction among others rapidly extended to plate guided waves (Lamb waves). Theoretical [4,5,6] and experimental [7,8,9] studies dealt firstly with band gap and waveguiding phenomena for air-solid and solid-solid phononic plates. Most of the work done so far for phononic plates restricts the sound wave propagation to the solid and the effect of the fluid (which could be treated as vacuum) is then neglected. Recently, phononic crystal strips are applied to enhance the quality factor of aluminum nitride (AlN) electroacoustic resonators utilizing the lowest symmetric (*S*_0_) Lamb wave mode thanks to strong reflection in acoustic band gaps [10].

On the other hand, a periodically perforated plate structure has been brought into focus while researching on the acoustic counterpart of Extraordinary Optical Transmission [11,12]. Resonant full transmission [13,14], Wood anomalies [15,16], and non-leaky modes [17,18] have been reported neglecting the role of the solid and constraining the wave propagation to the fluid. These two complementary points of view can be combined via fluid-solid coupling. Although more difficult to deal with, this unified perspective enables the observation of Extraordinary Sound Screening [19], complex interactions between Lamb-like modes and hole resonances [20,21], and full transmission through non perforated (corrugated) plates [22]. It is clear that the fluid-solid coupling plays a crucial role in the acoustical properties of phononic plates, portraying a wide range of different behaviours.

In this letter we provide experimental results for fluid loaded fluid-solid and air-solid phononic plates. Angle resolved transmission measurements of underwater ultrasound reveal key aspects of the different behaviour of phononic plates having fluid or air inclusions.

## 2. Materials and Methods

Underwater ultrasound has been chosen to measure the acoustic transmission through perforated plates. The geometrical parameters to describe perforated plates are the diameter of the hole d, the array periodicity a and the plate thickness h. The experimental setup is based on the well-known ultrasonic immersion transmission technique. This technique makes use of a couple of transmitter/receiver ultrasonic transducers. Two different couples of transducers were used: one couple with a center frequency of 250 kHz and a frequency range between 155–350 kHz and the other couple with center frequency of 500 kHz and a frequency range between 350 and 650 kHz. Each transducer was located at a distance larger than that of its nearfield distance (43 mm) from the plate and aligned with respect to the plate. A pulse is launched by the emitter piston transducer through the inspected plate. Then, the signal is detected by the receiving piston transducer and acquired by the pulser/receiver, post amplified and digitized by a digital PC oscilloscope (Picoscope model 3224, Cambridge, UK). Time domain data is finally analyzed after averaging 100 different measures and deleting unwanted reflections by means of a time window. The transmission spectrum is then calculated from the power spectrum of the signal normalized with the reference signal power spectrum measured without the sample plate. Typically, angle dependent measurements were done in angle steps of 1° and comprising from 0° to 60°.

The measurements were made using brass plates with 350 mm in width and 450 mm in length (ρ = 7890 kg/m^3^, *c*_l_ = 5670 m/s, *c_t_* = 3230 m/s) and 2 mm thickness, immersed in water (ρ = 1000 kg/m^3^, *c*_l_ = 1480 m/s).

**Figure 1:**
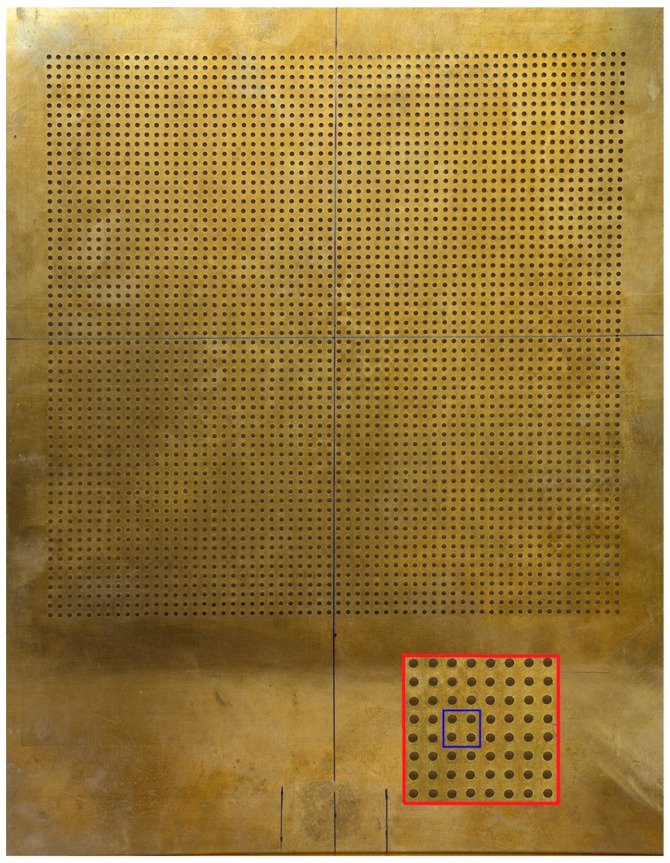
Structure and elementary cell for a brass plate perforated with periodically square distribution of circular holes.

A brass plate drilled with periodically square distribution of circular holes having diameter, *d*, of 1 mm and a unit cell period, *a*, of 3 mm, was used (Figure 1). Starting from the perforated plate the fabrication of an air-solid phononic plate was carried out by sticking, carefully, a film (20 µm thick) around the plate, taking care that did not appear undesired air bubbles when the film was adhered.

## 3. Results and Discussion

**Figure 2:**
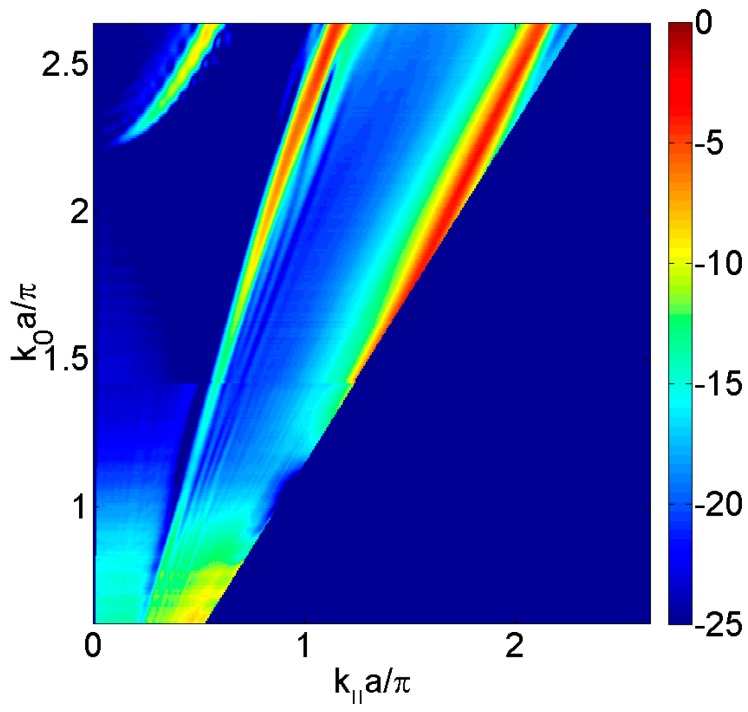
Measured transmitted sound power coefficient, τ, as a function of the parallel wavevector *k_‖_*·*a*/π in the Γ*X* direction and the normalized frequency *k*_0_·*a*/π, where *a* the array periodicity. The transmitted sound is represented in dB scale.

When an incident sound pressure wave impinges on the plate, gives rise a reflected and a transmitted sound pressure wave. In the solid, high transmission values can be observed revealing a complex dispersion. For this case, three kinds of modes can be distinguished: Scholte-Stoneley mode, symmetric leaky Lamb modes, and antisymmetric leaky Lamb modes. The Scholte-Stoneley mode propagates across the fluid-solid interface with a phase speed slightly slower than that of the water. At low frequencies, this mode is mixed with the *A*_0_ mode and slowly converges to the sound line as ω increases. Leaky Lamb modes are guided waves produced due to the multiple reflections of longitudinal and in-plane transverse modes at both plate-fluid interfaces. Figure 2 shows the different shapes of the symmetric and antisymmetric modes in a 2 mm thickness brass plate. Although there was not periodicity due to it was not still drilled, in order to compare with subsecuent results, these are plotted in terms of the normalized frequencies *k*_0_·*a*/π and *k_‖_*·*a*/π, where *a* is the array periodicity of the perforated plate, where *k*_0_ is the incident wavenumber and *k_‖_* is *k*_0_ projection on the plate.

**Figure 3:**
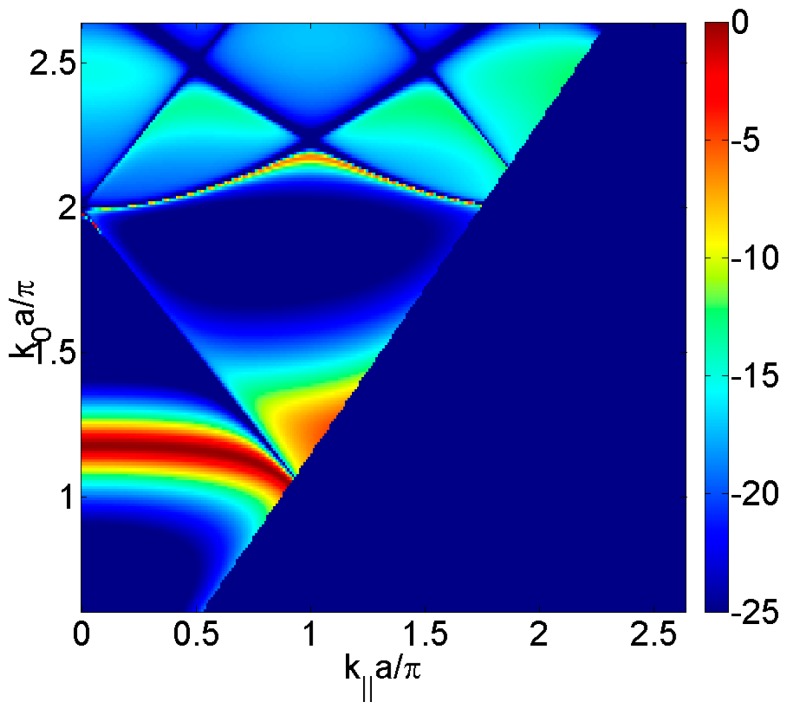
Calculated transmitted sound power coefficient, τ, as a function of the parallel wavevector *k_‖_*·*a*/π in the Γ*X* direction and the normalized frequency *k*_0_·*a*/π, for the square distribution of circular holes having diameter, *d*, of 1 mm and a unit cell period, *a*, of 3 mm. The transmitted sound is represented in dB scale.

However, when the plate is periodically perforated, one has to consider the elastic movement of the plate coupled with the surrounding fluid not only at the plate free surface but also inside the apertures. In order to evaluate the physical phenomena, without taking into account the elastic movement of the plate, the transmission sound power can be calculated by solving the sound wave equation within a hard-solid model [23], that is, assuming that the pressure field does not penetrate into the material plate because the water/plate impedance is considered infinite. Figure 3 shows the calculated transmitted sound power coefficient, τ, for the periodically square distribution of circular holes considered and it is observed the Fabry-Perot full transmission at *k*_0_·*a*/π = 1.2 and the Wood anomaly minima at *k*_0_·*a*/π = 2 that is evident when the incidence angle is varied. Due to the elastic movement of the plate had not been taken into account, Lamb and Scholte-Stoneley waves are not observed.

**Figure 4:**
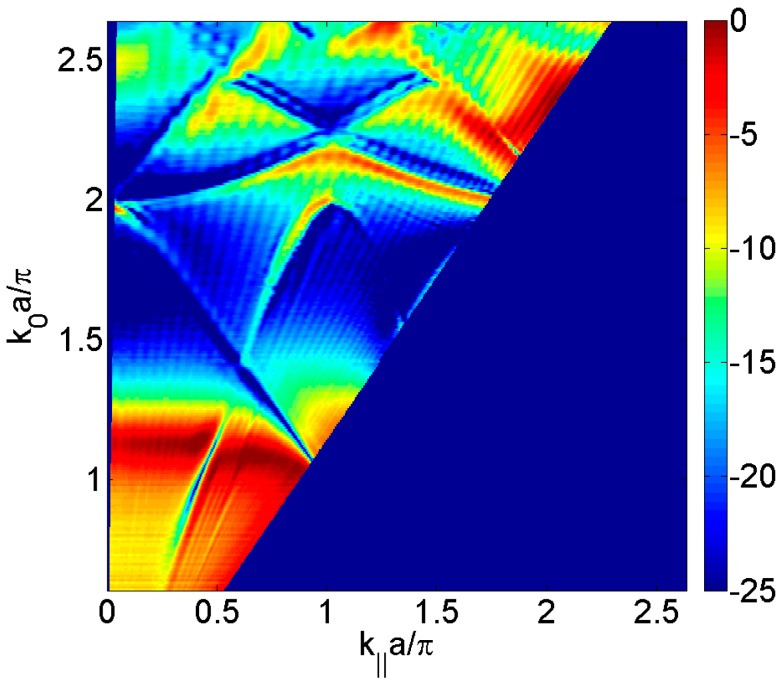
Measured transmitted sound power coefficient, τ, as a function of the parallel wavevector *k_‖_*·*a*/π in the Γ*X* direction and the normalized frequency *k*_0_·*a*/π, for the square distribution of circular holes having diameter, *d*, of 1 mm and a unit cell period, *a*, of 3 mm. The transmitted sound is represented in dB scale. The transmitted sound is represented in dB scale.

Figure 4 depicts the experimental transmitted sound power τ, as a function of the parallel wavevector *k_‖_*·*a*/π in the Γ*X* direction and the normalized frequency *k*_0_·*a*/2π, of the brass plate perforated of thickness *h* = 2 mm with holes of diameter *d* = 1 mm arranged periodically in a square lattice of period *a* = 3 mm and immersed in water. Complex interaction between minima and maxima is present in the spectra and it makes clear that the symmetry of the array results in a high angular dependence of the spectra. For *k_‖_*·*a*/π near zero (normal incidence) the Fabry-Perot full transmission at *k*_0_·*a*/π = 1.2 and Wood anomaly can be clearly seen both thickness at *k*_0_·*a*/π ≈ 2. However, the modes appearing from the bottom of the figures are not predicted by the Wood anomaly and are related to leaky surface modes. These surface modes are leaky Lamb modes observed in the homogeneous plate (see Figure 2) arising from the plate vibration and the solid-fluid coupling.

**Figure 5:**
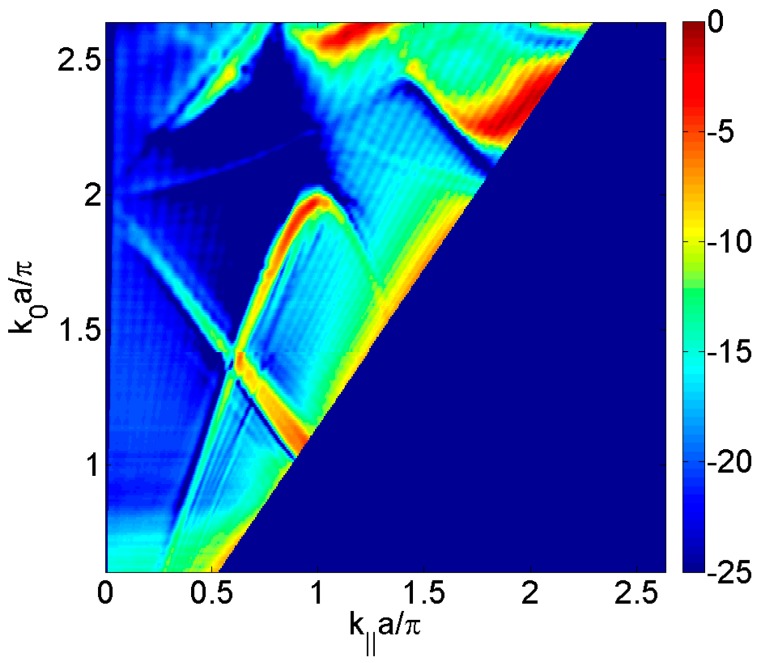
Measured transmitted sound power coefficient, τ, as a function of the parallel wavevector *k_‖_*·*a*/π in the Γ*X* direction and the normalized frequency *k*_0_·*a*/π, for the square distribution of circular holes having diameter, *d*, of 1 mm and a unit cell period, *a*, of 3 mm and the holes filled with air (air-solid phononic plate). The transmitted sound is represented in dB scale.

The experimental dispersion of the same brass plate but having air filled holes is showed in Figure 5. As the wave interaction at an air-solid interface is different from that of a fluid-solid interface, the transmission properties of the phononic plate differ from the previous case. As the holes have been filled with air, the Fabry-Perot resonance was highly attenuated due to the impedance difference between water and air. Zero order Lamb modes, Scholte-Stoneley mode near grazing incidence and Wood anomalies are clearly distinguishable but there are new transmission modes that have not been detected in the case of the water filled holes.

**Figure 6:**
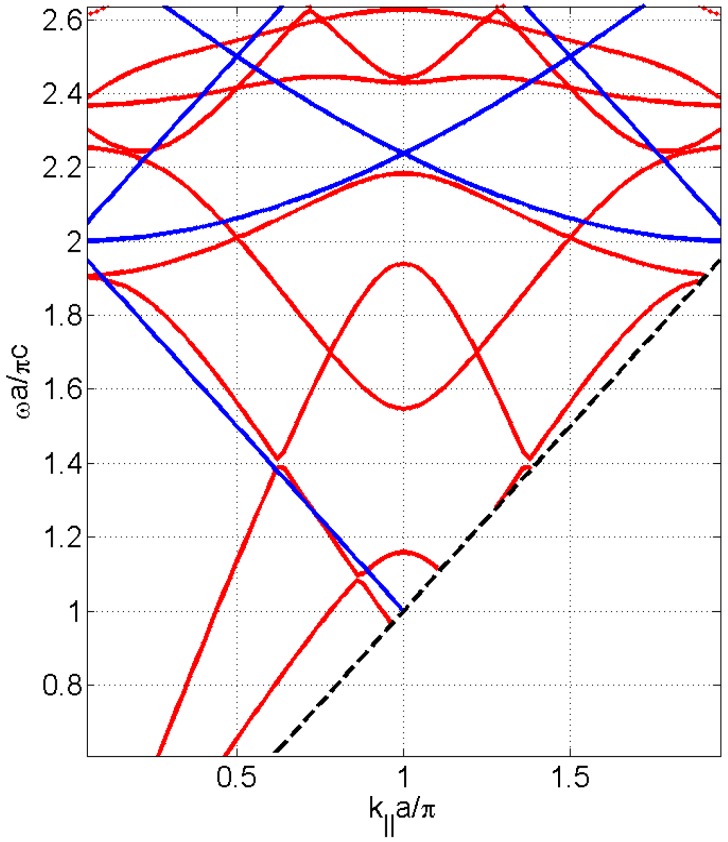
Calculated dispersion diagram (red lines) and Wood minima (blue lines), as a function of the parallel wavevector *k_‖_*·*a*/π in the Γ*X* direction and the normalized frequency ω*a*/πc, for the square distribution of circular holes having diameter, *d*, of 1 mm and a unit cell period, *a*, of 3 mm.

The nature of these transmission modes can be demonstrated by calculating the dispersion diagram for the brass phononic plate. For this purpose we calculate the eigenfrequencies of the cell type with periodic boundary conditions of Floquet type with a variable vector $\vec{k}=(k,0,0)$ between 0 and π/2*a*. Figure 6 shows the dispersion diagram.

**Figure 7:**
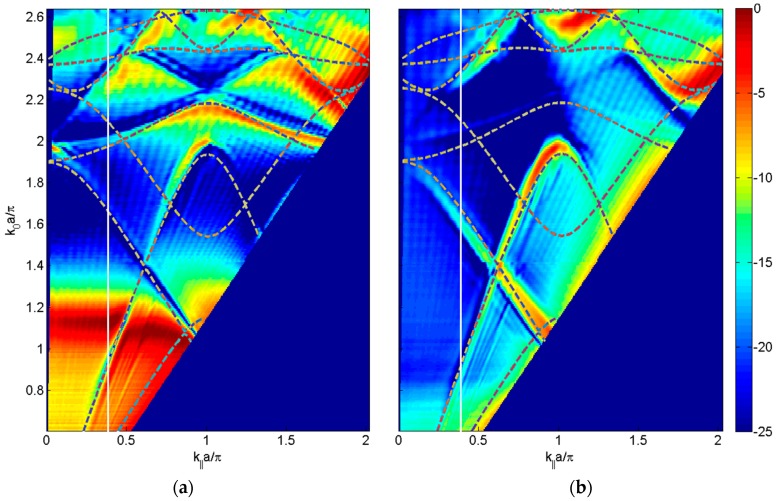
Dispersion diagram superimposed to measured transmitted sound power coefficient, τ, as a function of the parallel wavevector *k_‖_*·*a*/π in the Γ*X* direction and the normalized frequency *k*_0_·*a*/π, for the square distribution of circular holes having diameter, *d*, of 1 mm and a unit cell period, *a*, of 3 mm and the holes filled with (**a**) water and (**b**) air.

By superimposing the dispersion diagram with the graphics of the transmitted sound power coefficient as a function of the parallel wavevector for the perforated plates with water or air-filled holes (see Figure 7), it is observed that while some lines of the dispersion diagram clearly indicate a significant increase in transmission through plate, others do not exhibit.

**Figure 8:**
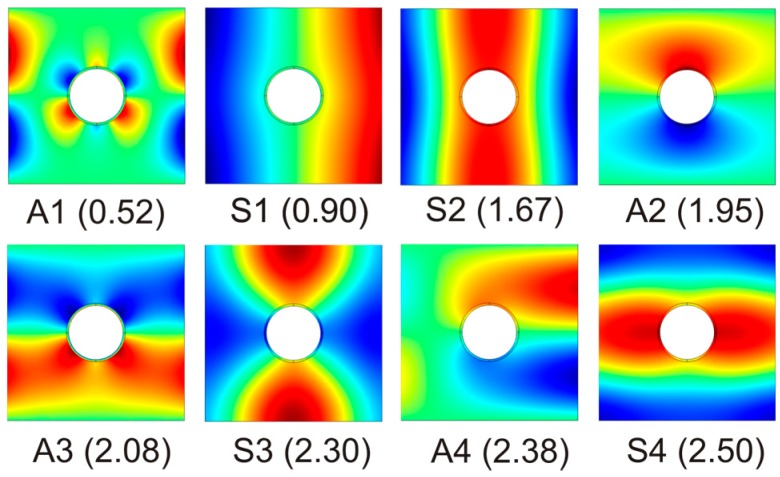
*z*-displacement of the brass unit cell for a fixed value of *k_‖_*·*a*/π = 0.4 The eigenmodes are labeled as *S* (symmetric) or *A* (antisymmetric) according to they behaviour to the horizontal plane wave incoming from the left side of the plots. The eigenmodes normalized frequency *k*_0_·*a*/π values are despited in parenthesis. The displacement is represented in linear scale.

To analyze this fact, we calculate the displacement of the unit cell in the *z* direction for a fixed value of the parallel wavevector *k_‖_*·*a*/π for the corresponding eigenfrequency. Figure 8 shows some eigenmodes for *k_‖_*·*a*/π = 0.4. It is noted that some are antisymmetric modes (*A*_1_, *A*_2_, *A*_3_ and *A*_4_) with respect to the forward direction of the plane wave. Clearly these eigenmodes produce poor fluid-solid coupling and lead to non-radiative bands, while symmetric eigenmodes (*S*_1_, *S*_2_, *S*_3_ and *S*_4_) produce defined transmission lines.

## 4. Conclusions

The results presented in this letter give a wide description of the phenomena involved in phononic plates as they also includes the coupling with the surrounding fluid. The fluid-solid phononic plates behavior is mainly governed by holes resonances and coherent diffraction effects which interact with Lamb-like modes. However, in air-solid phononic plates, zero order Lamb modes, Scholte-Stoneley mode near grazing incidence, Wood anomalies and new transmission modes are clearly distinguishable while the Fabry-Perot resonance was highly attenuated due to the impedance difference between water and air. It has been reported here that these differences are due to antisymmetric eigenmodes produce poor fluid-solid coupling and lead to non-radiative modes, while symmetric eigenmodes has high fluid-solid coupling and lead radiative modes.
